# First report of autochthonous non-vectorial canine leishmaniasis in New Caledonia, south-western Pacific: implications for new control measures and recommendations on importation of dogs

**DOI:** 10.1186/s13071-016-1388-6

**Published:** 2016-02-25

**Authors:** Nathalie Daval, Céline Marchal, Laurent Guillaumot, Thomas Hüe, Christophe Ravel, Nicolas Keck, Mohamed Kasbari

**Affiliations:** Cabinet Vétérinaire du Regain, Nouméa, Nouvelle-Calédonie; Service des Laboratoires Officiels Vétérinaires, Agro-alimentaires et Phytosanitaires de la Nouvelle-Calédonie, LNC-DAVAR, Païta, Nouvelle-Calédonie; Institut Pasteur de Nouvelle-Calédonie, IPNC, Nouméa, Nouvelle-Calédonie; Institut Agronomique néo-Calédonien, « Connaissance et amélioration des agrosystèmes », Laboratoire de Parasitologie, Païta, BP 73, 98890 Nouvelle-Calédonie; Département de Parasitologie-Mycologie—CHU de Montpellier, Centre National de Référence des Leishmanioses, Montpellier, France; Laboratoire Départemental Vétérinaire de l’Hérault, Montpellier, LDV 34 France; Agence Nationale de Sécurité Sanitaire de l’Alimentation, de l’Environnement et du Travail, ANSES, Laboratoire de Santé Animale, 14 rue Pierre et Marie Curie—94701, Maisons-Alfort, Cedex France

**Keywords:** Canine leishmaniasis, New Caledonia, Importation risk, Phlebotomine sandflies, Non-vectorial transmission, Transplacental and venereal transmission routes

## Abstract

**Background:**

Canine leishmaniasis (CanL), a parasitic zoonotic disease caused by *Leishmania infantum* and usually transmitted by phlebotomine sandflies, has rarely been reported in Pacific islands, which have been regarded until now as leishmaniasis-free territory. Here, we report the first autochthonous CanL case in New Caledonia (south-western Pacific) and the investigations carried out 1) to determine how infection was introduced into and transmitted among these dogs and 2) to assess the risks to animal and public health.

**Methods:**

Extensive epidemiological and entomological investigations in and around the focus were carried out. Leishmaniasis infection was confirmed by histopathology, indirect fluorescent antibody test, real-time PCR, and culture. Parasite strain was typed by the isoenzymatic technique.

**Results:**

The survey revealed close contacts between the autochthonous dog and two infected bitches imported from Spain, but failed to find any possible vector or disease spreading to other animals or humans. *L. infantum* zymodeme MON-1, the most frequent type in the Mediterranean basin, was identified. Although transplacental and venereal transmissions could not be excluded, the evidence was in favour of non-vectorial, direct dog-to-dog transmission.

**Conclusions:**

This study corroborates the possibility of non-vectorial routes (transplacental, venereal, and direct dog-to-dog) of canine leishmaniasis transmission in New Caledonia and raises the debate of relevant test requirements and diagnostic sensitivity prior to importation of dogs in *Leishmania*-free regions. New leishmaniasis control measures and recommendations to avoid future CanL introduction on the island are discussed.

## Background

Canine leishmaniasis (CanL) is a vector-borne disease, caused by *Leishmania infantum*, an intra-cellular protozoan parasite. It is an important worldwide zoonosis, endemic in approximately 98 countries mainly in the Mediterranean region, Africa, Southern Asia, and Latin America. The parasite is commonly transmitted through the bite of a female phlebotomine sandfly, but alternative modes of transmission have often been reported in the literature [1–3].

New Caledonia is an island country, a special collectivity of France located in the south-west Pacific Ocean, 1200 km east of Australia and 1500 km north of New Zealand. The main island comprises an elongated mountainous range surrounded by coastal plains with a tropical climate. The population of approximately 250,000 is concentrated around the capital, Noumea.

New Caledonia was officially declared free of canine leishmaniasis by OIE World Organisation for Animal Health, and both the Pasteur Institute and Sanitary Services (DASS-NC) of New Caledonia confirm that no human cases have ever been detected in the country to date (authors’ personal communication). Although phlebotomine sandflies species have been reported since 1921 in the Australasian region [4] and 33 species have been described in New Zealand and Australia [5, 6], there is only one record of the presence of phlebotomine sandflies, which belongs to the genus *Australophlebotomus*, in New Caledonia [7] and no proven or suspected vector of *Leishmania* has ever been reported to date in New Caledonia or in other surrounding Pacific islands.

As a systemic disease that can involve any organ, tissue or body fluid, CanL displays a wide range of clinical expression. Lymphadenomegaly, loss of body condition, pale mucous membranes, splenomegaly, alopecia, furfur, and onychogryphosis are the most frequently observed clinical signs [8–10] but many other clinical features, alone or combined, like polyuria/polydipsia, diarrhea, fever, ocular signs, or arthropathy can be present. However, most dogs remain asymptomatic despite a confirmed infection. Consequently, the CanL diagnosis is challenging for clinicians, and need laboratory confirmation, especially in a leishmaniasis-free territory where no transmission is supposed to occur.

In this study, we describe the first autochthonous case of CanL in New Caledonia, and the investigations carried out to determine how infection was introduced into and transmitted among the dogs. The hypotheses raised to explain the transmission mode at work and the risks to animal and public health in New Caledonia are discussed, and recommended leishmaniasis control measures to avoid new introduction on the island are proposed.

## Methods

**Fig. 1:**
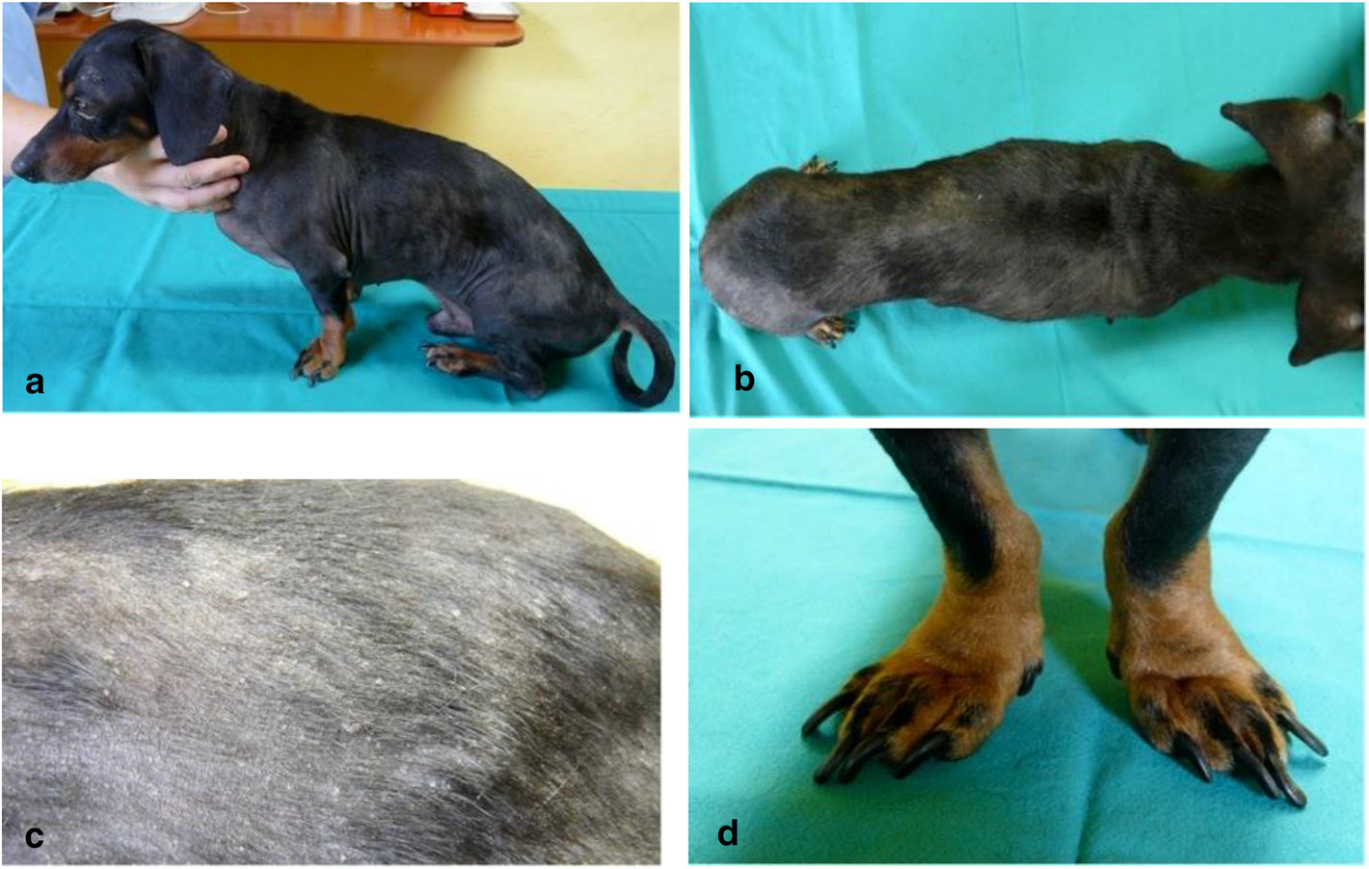
*Leishmania infantum*-infected dachshund: **a** General lateral view. **b** Extreme slimness. **c** Diffused alopecia and exfoliative dermatitis. **d** onychogryphosis

A 4-year-old male dachshund, born in New Caledonia in September 2006 and without overseas travel history, was presented to a veterinary clinic in Dumbea in November 2010, with a history of weight loss, non-pruritic exfoliative dermatitis, alopecia, onychogryphosis (Fig. 1), bilateral conjunctivitis, skin and gingival mucosa ulcerative lesions without lymphadenomegaly. The typical onychogryphosis, old-dog face, and furfur suggested a *Leishmania* infection. Nevertheless, some of the clinical signs were similar to those reported in other canine vector-borne diseases (CVBDs), especially babesiosis, ehrlichiosis or anaplasmosis. However, although New Caledonia is not tick-free, ticks of mammals are a later introduction into the island and no canine disease transmitted by ticks was declared autochthonous or enzootic to date. The rare CVBDs cases are considered as imported infections. Besides, the dachshund was correctly treated against ectoparasites, the owner didn’t mention any tick bites and stained blood smears for parasite detection were negative. To achieve an etiological diagnosis, firstly, we performed skin biopsy and haematological and biochemical blood analysis, whose results, secondly, guided appropriate serological and molecular test selection.

### Skin histopathology, serology and PCR

Four skin biopsies formalin-fixed, paraffin-embedded and stained with haematoxylin-eosin were collected for histopathological examination. Serological testing for *Leishmania* was carried out with the in-house indirect immunofluorescence antibody technique (IFAT), counter immunoelectrophoresis (CIE) and a commercial enzyme-linked Immunosorbent assay (Elisa) kit (Bordier Affinity Products, Switzerland). TaqMan-based kinetoplastic DNA minicircle targeted real-time polymerase chain reaction (RT-PCR) was performed on whole blood and skin samples as previously described [11].

### *Leishmania* isolation and strain typing

Skin, lymph nodes, bone marrow, and spleen tissue samples were collected and cultured at 25 °C for 2 months in RPMI with 2 mM L-glutamine and Dulbecco’s Modified Eagle Media (DMEM) without L-glutamin, supplemented with 20 % of foetal calf serum, 3 % of HEPES, and 50 IU/ml penicillin-streptomycin. *Leishmania* strain isoenzymatic typing was performed as described previously [12]. Moreover, DNA sequence coding for putative translation initiation factor eIF-2B alpha subunit gene, was amplified, sequenced, and identified by Blast search.

### Epidemiological investigations

The pedigree, origin, and complete travel history of the infected dachshund were requested and a zone around the CanL focus was investigated to find other possible cases. Veterinary clinics and dog owners were surveyed. To detect possible intercontamination and CanL spread, all pets sharing the same household inside the focus were tested with IFAT, ELISA, CIE and PCR. Nine people exposed to close and frequent contact with the infected dachshund were also serologically tested for *Leishmania* infection.

### Entomological survey

**Fig. 2:**
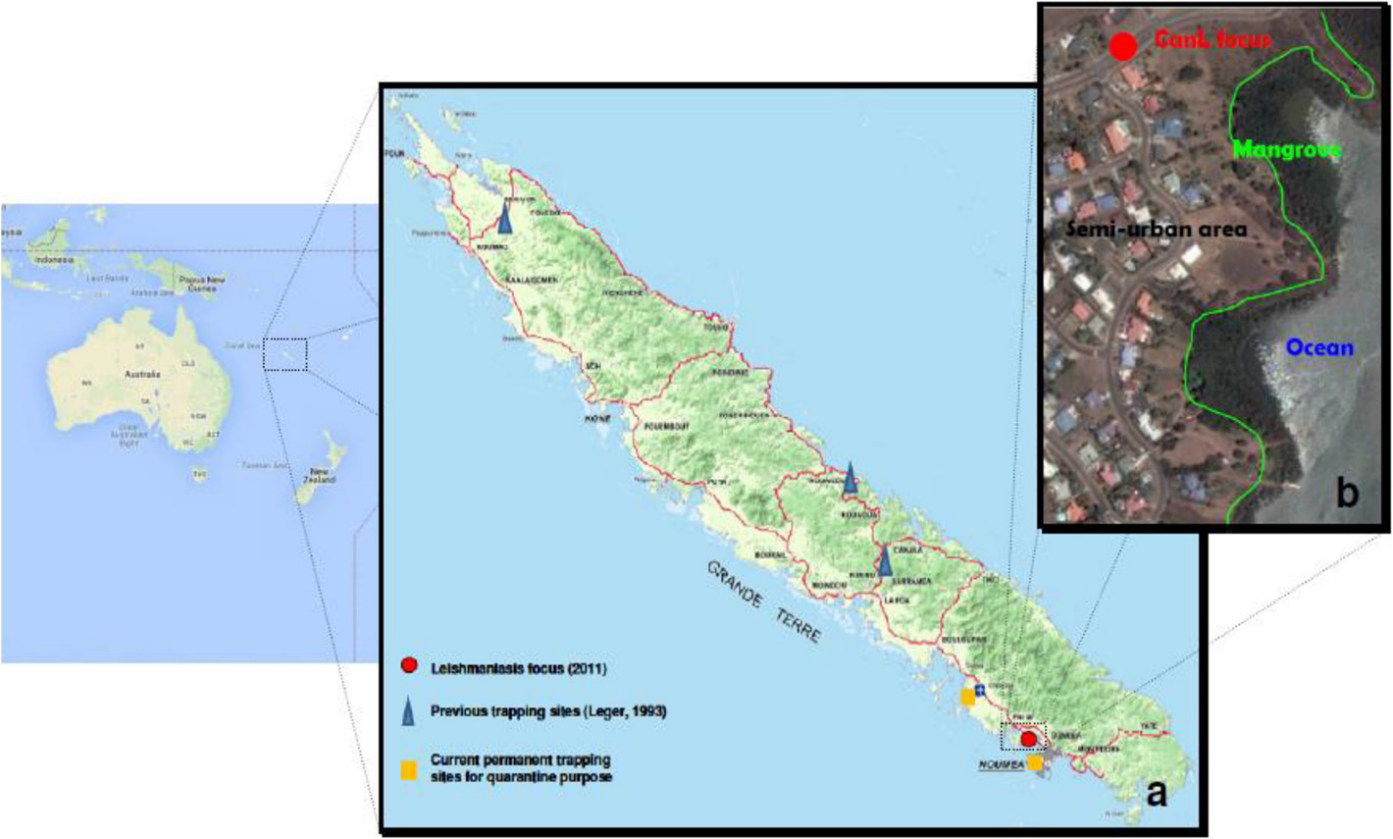
Map of New Caledonia showing: **a** The canine leishmaniasis focus and entomological trapping sites locations. **b** Aerial view of the semi-urban area around the canine leishmaniasis focus, surrounded by mangrove swamp

Specific entomological investigations in the residential area of the index case were carried out for 3 weeks from 4 February to 16 February 2011. CDC Miniature Light Traps (John W Hock Co., Gainesville, FL, USA) with black light and with white light, with and without CO_2_ (dry ice), BG Sentinel® (Biogents AG, Regensburg, Germany) traps with and without CO_2_, and paper sheets impregnated with castor oil were placed in the afternoon on dog owners’ property and retrieved 24 h later. The trap locations included sheltered and unsheltered places, and the papers were placed mainly at the two entrances of a shelter. Insect trapping targeting mainly mosquitoes is usually carried out all year round in New Caledonia for both vector surveillance and quarantine purposes with CDC Miniature Light Traps with black light and CO_2_, in the surroundings of seaports, airports, and industrial, residential and rural areas. These traps are equipped with fine mesh bags which can also retain small insects such as phlebotomine sandflies. Since the report of this case, special care has been taken to trap sandflies (Fig. 2).

### Ethics statement

All animals were handled in accordance with good animal practices required by the European and French ethical guidelines and animal welfare regulations (Directive 2010/63/EU). Moreover, the dogs’ owner, the human participants, the local new Caledonian health, veterinary and administrative authorities, were comprehensively informed about the study, and oral consents were obtained.

## Results

### Laboratory analyses

A complete blood count revealed hyporegenerative anaemia with mild reticulocytosis, leukopenia with lymphopenia and thrombocytopenia. Serum biochemical analysis highlighted a hyperproteinemia and a hypoalbuminemia (without proteinuria), due to chronic inflammation and hyperglobulinemia. However, uremia, creatininemia and liver enzymes levels were in the normal reference range and neither renal nor liver failure were detected.

**Fig. 3:**
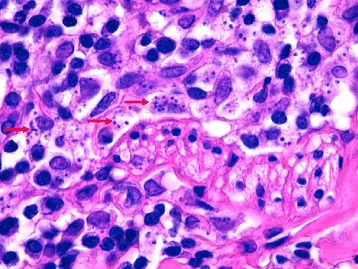
Skin biopsy of cutaneous leishmaniasis, H&E stained, x 100: Numerous intracytoplasmic *Leishmania sp*. (*red arrows*) visible within macrophages, (A. Poujade, LAPVSO, France)

The skin histopathological examination showed lichenoid granulomatous nodular parasitic dermatitis consistent with cutaneous leishmaniasis (Fig. 3). Serum displayed positive results with IFAT (titre 1/1280) and counter immunoelectrophoresis (CIE), suggesting an evolving leishmaniasis infection, whereas RT-PCR was positive for whole blood and skin samples. *Leishmania* promastigotes were isolated from all tissue samples and isoenzymatic typing identified *L. infantum* zymodeme MON-1, the most frequently encountered type in humans and dogs in Mediterranean countries. Factor eIF-2B alpha subunit gene sequencing showed 100 % identity with *L. infantum* reference sequence.

Because of the dog’s poor clinical condition, the confirmed infection, and the leishmaniasis-free status of New Caledonian territory, euthanasia was requested by the Official Veterinary Services and the CanL focus was notified to OIE.

### Epidemiological investigations

**Fig. 4:**
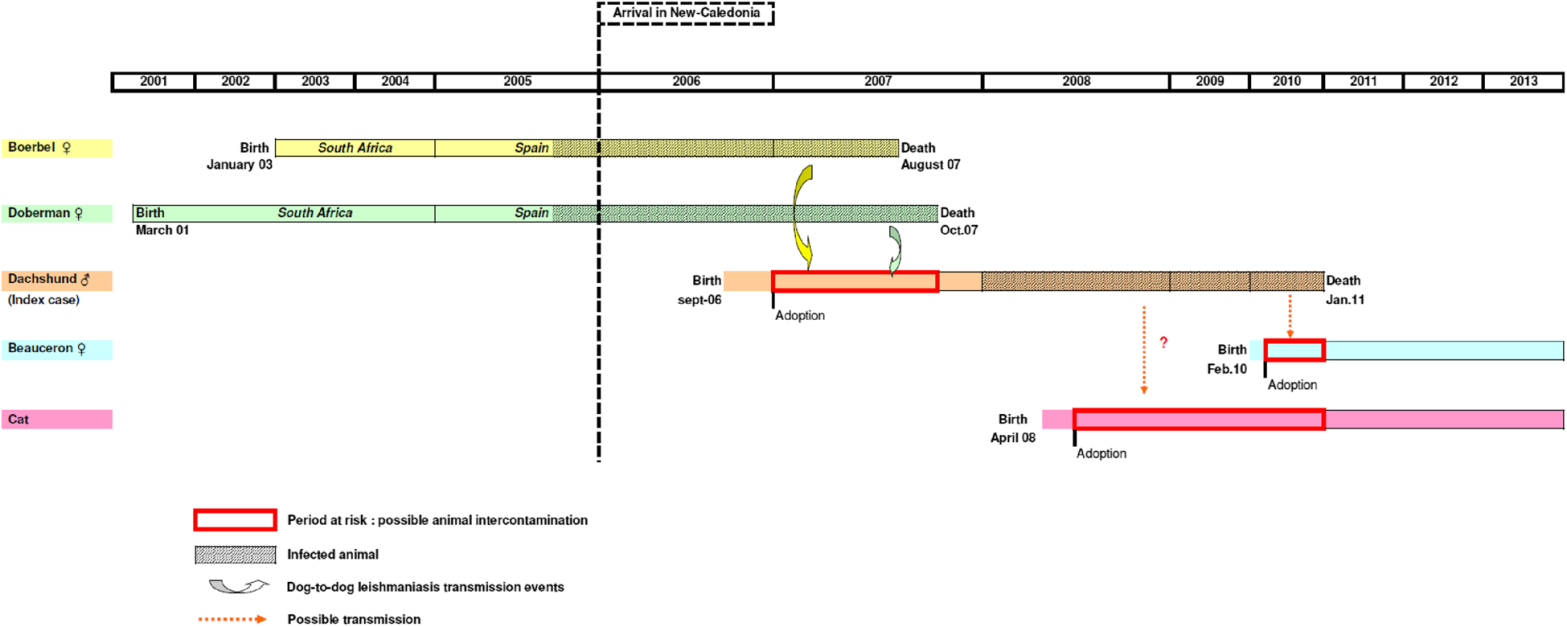
Timeline showing pet introduction, infection events, and possible animal intercontamination within peer group

The retrospective epidemiological survey revealed that the infected dachshund was born in New Caledonia, with no history of travelling abroad, and was raised for the first year with two female dogs, a Dobermann and a Boerboel, that had previously spent 2 years in South Africa and then 1 year in Spain before settling in New Caledonia in 2006 with their owners (Fig. 4). The Boerboel bitch presented with lymphadenomegaly and died in August 2007 at the age of four and a half years, when the dachshund was 11 months old. A multicentric lymphoma was suspected but neither histologic nor cytologic confirmatory analyses were performed. In October 2007, the Dobermann bitch also died after a short period of severe apathy and presumed renal and cardiac failure without aetiological investigation. The owner reported that, at that time, the young male dachshund used to play frequently with the two bitches. However, no mating was observed and the young age and small size of the dachshund compared with the females didn’t support a mating event, although this cannot be totally excluded.

Moreover, from May 2008 to the end of March 2010, a cat and another young Beauceron bitch, 2 months old, were adopted and shared the same household as the infected dachshund (Fig. 4). In order to detect possible intercontamination and CanL spread, these two pets were monitored every 3 months for a year by clinical examination, serology (IFAT, CIE, and ELISA) and RT-PCR on blood samples. All results were negative, except for the cat which showed, once, a low IFAT antibody titre (1/160) decreasing within 4 months without any treatment (Table 1). The nine people exposed to contact with the infected dachshund were all confirmed to be seronegative.

**Table 1:** Serological and PCR monitoring of the index case peers

Date	Beauceron dog		Cat	
	Serology	PCR	Serology	PCR
23 Feb. 2011	IFAT: Negative	Negative	/	Negative
22 Jun. 2011	IFAT: 1/20	Negative	/	Negative
	CIE: Negative			
6 Sep. 2011	IFAT: 1/20	Negative	/	Negative
2 Feb. 2012	IFAT: Negative	Negative	IFAT: 1/160	Negative
9 Feb. 2012	/	/	IFAT: 1/80	Negative
			CIE: non-specific reaction	
7 Mar. 2012	/	/	IFAT: 1/80	Negative
			ELISA: Negative	
			CIE: non-specific reaction	
19 Jun. 2012	/	/	IFAT: 1/20	Negative
			CIE: Negative	

*CIE* counterimmunoelectrophoresis

### Entomological survey

Several species of *Culicidae*, *Ceratopogonidae* and *Psychodinae*, but no *Phlebotominae*, were caught in the area of the index case. The Insect trapping program mentioned here-above, carried out all year round for mosquito surveillance in New Caledonia, did not show any presence of phlebotomine sandflies either so far.

## Discussion

The first autochthonous canine leishmaniasis case in New Caledonia, described herein, raises several issues about: 1) the origin of this *Leishmania* parasite; 2) the route of autochthonous dog contamination; 3) the risk level of disease spreading to local animal or human populations; and 4) the control measures that should be established to avoid the spread of leishmaniasis and any further leishmaniasis introduction in New Caledonia.

The introduction of *Leishmania*-infected dogs from an endemic area to a leishmaniasis-free island or territory has often been observed: in Japan, one case was imported from Spain [13], and two cases from Sicily in 2006 [14]. In the United Kingdom, more than 250 imported leishmaniasis cases were reported between 2005 and 2007 [15]. Most of the infected dogs had spent several months in endemic countries such as Spain, Portugal, Greece, Italy or France. In Australia, a retrospective case study of five imported dogs diagnosed with CanL between 2000 and 2011 highlighted the importance of obtaining a travel history [16].

In New Caledonia, only 18.6 % of dogs and cats admitted after quarantine to the island in 2013 originated from *Leishmania*-free countries (mainly New Zealand, Australia and Tahiti) and most (81.4 %) came from France, where canine leishmaniasis is endemic in the southern regions.

In our case study, the epidemiological survey revealed the introduction in New Caledonia of two female dogs from Spain which displayed clinical signs consistent with leishmaniasis and lived for 8 to 10 months, until they died, with an autochthonous male dachshund which could have been exposed to the infection during this contact period (Fig. 4). Indeed, this dachshund had never been imported from, or taken to, an endemic region. In addition, autochthonous animal leishmaniasis infections have never been previously recorded in New Caledonia.

Several hypotheses can be considered to explain the mode of contamination. The most common route of leishmaniasis transmission is sandfly bites.

Only two phlebotomine species have been reported in New Caledonia, and belong to the genus *Australophlebotomus*: *A. maduloae* and *A. nottheghemae* [7]. The species within this genus are not known to be competent vectors for *Leishmania spp*. which is reputedly transmitted by species of the genera *Phlebotomus* (Old World) or *Lutzomyia* (New World) [17], although the possible role of some *Sergentomyia* species is being viewed with increasing suspicion, at least regarding *Leishmania* transmission within animal populations [18, 19]. No species belonging to any of these three genera have ever been reported on New Caledonian territory. The two local species described by Leger and Pesson [7] were captured in the mountain range in central areas of the island (both species) and on the eastern coast (*A. notteghemae*) (Fig. 2). In all cases, these sandflies were captured in wild environments close to caves, whereas the infected dog was living in a sparsely populated but urban area surrounded by coastal mangrove swamp, where no phlebotomine sandflies have ever been reported. Therefore, because of their geographical distribution, weak population densities, exophilic behaviour, natural cave habitats, and the limited number of CanL cases, the implication of the local phlebotomine species in the leishmaniasis transmission, inside the studied focus, seems very unlikely. The hypothesis of a newly introduced *Leishmania*-competent *phlebotomine* species in New Caledonia is also very unlikely because of the bionomics of these fragile insects which is poorly consistent with overseas transportation at any stage, and not supported by the negative results of the entomological surveillance carried out all year round on the island under the mosquito control program. The risk of classical vectorial leishmaniasis spreading on the island is therefore, according to current knowledge, very low. Nevertheless, further studies to determine the competence of *Australophlebotomus* species for *Leishmania* transmission are needed.

Regarding Leishmaniasis transmission by other vectors such as ticks or fleas, which has sometimes been suggested [20, 21], although such arthropods are present in the infected dog’s environment, full development and transmission of *Leishmania* metacyclics formed through bites have never been demonstrated in these arthropods [22]. Besides, the dachshund was regularly treated against external parasites with Fipronil (Frontline, Merial). Hence, such a transmission route can be discarded.

Hitherto, only *Forcipomyia sp*., a day-feeding midge of the subgenus *Lasiohelea* (*Ceratopogonidae* family) has been strongly incriminated as a competent vector for a new exotic *Leishmania* species reported in Australia and implicated in kangaroo leishmaniasis infection [23]. Nevertheless, although the *Forcipomyia* genus is distributed worldwide and its presence on New Caledonian territory is likely, multiple cases or endemicisation of leishmaniasis have not, until now, been observed by clinicians in New Caledonia as they were among macropods in the Northern Territory of Australia [24]. Moreover, the molecular and isoenzymatic typing of *Leishmania* isolated from the infected dachshund identified the parasite as *Leishmania infantum*, MON-1, commonly reported in the Mediterranean basin.

Consequently, the most likely option remains a non-vector-borne transmission that could be venereal, transplacental or direct from dog to dog.

Venereal transmission of canine leishmaniasis has been well described in the literature [3, 25–27] but is here unlikely considering the age and size differences between the three dogs at the time of their cohabitation: the diagnosis was based on a young dachshund (Teckel purebred group, 20 cm at the withers) which was less than 1 year old when living with the two suspected adult bitches imported from Spain, a Boerboel and a Dobermann, 60 and 65 cm at the withers respectively. The owner also did not mention any mating of the young dachshund. Nevertheless, this possibility cannot be completely excluded.

Concerning transplacental transmission, evidences have been regularly reported [2, 28–30] and the current debate about the vertical route of transmission is now focused mainly on its frequency, real efficiency, and epidemiological contribution to the disease cycle. In this study, data about the autochthonous dog’s pedigree and parents’ leishmaniasis status were not available. Hence, transplacental transmission cannot be totally ruled out despite the *Leishmania*-free status of New Caledonia.

Contamination through needles or transfusion is unlikely as the owner never used needles for any treatment and the infected dog had never received a blood transfusion.

Thus, a likely infection route of the dachshund remains contamination through direct dog-to-dog contact. Indeed, the two imported dogs (Dobermann and Boerbel bitches) died of unidentified pathologies but showed clinical signs consistent with CanL. Moreover, both of them spent 1 year in Spain, a country where leishmaniasis is highly endemic, just before their arrival in New Caledonia. At that time, no analysis targeting leishmaniasis was carried out on these animals.

Direct dog-to-dog transmission has also been suspected [31] but not yet confirmed by experimental evidence [32]. Nevertheless, direct transmission through licking and biting behaviours with exchanges of body fluids remains possible. The autochthonous infected dachshund lived, from puppy to young dog stage, in close contact for approximatively 10 months with the Boerbel and for 1 year with the Dobermann dog, at the precise time when both were ill (Fig. 4). Moreover, typical young dog fight games as reported by the owners, are consistent with this transmission mode.

The dachshund’s new congeners, a Beauceron dog and a cat, were followed up by clinical examination, IFAT, Elisa, CIE and PCR over an 18-month period. Regardless of the technique used, the Beauceron dog proved negative. Cat sera showed a low ambiguous IFAT antibody titre decreasing within 4 months while Elisa and PCR were negative and counter immunoelectrophoresis displayed a non-specific precipitin line (Table 1). Cross-reactions between feline antibodies to different *Leishmania* and *Trypanosoma* species as also shown in dogs [33] or, possibly, to *Toxoplasma gondii* [34] have already been reported. Also, the determination of serological cut-off titres with standardised protocols and definition of a gold standard technique as a routine laboratory test validated for cat species, is still required, especially with low reactive sera. Consequently, our aggregate cat analysis results and epidemiological context (autochthonous cat born in New Caledonia without travel to endemic country or close contact with infected dog) argue here for a feline non-specific antibody response probably owed to cross-reaction with other trypanosomatid or pathogen infections.

Therefore, a vectorial leishmaniasis spread from the index case to local animal or human populations is, with the current knowledge, very unlikely. However, the risk of leishmaniasis becoming established within New Caledonia through a non-vectorial mode with new importations of infected animals should be seriously considered. Indeed, in the USA [32, 35] and in France (author’s personal communication) canine leishmaniasis was slowly disseminated through kennels in non-endemic areas, in the absence of known competent vectors, through venereal and transplacental transmissions.

### Dog importation: CanL control measures and recommendations

Subsequent to this case, an awareness campaign about canine leishmaniasis was launched to remind veterinary clinicians of clinical signs, diagnostic, current regulations and recommendations. Financial support for laboratory analysis was set up for any suspect case of CanL. Moreover, dogs and canine semen importation regulations in New Caledonia were strengthened: the imported dogs must be subjected to an IFA or ELISA or western blot test to detect *Leishmania infantum* antibodies, with a negative result, within 30 days prior to the date of shipment. Although positive *Leishmania* PCR on semen has been reported [3, 25] and culture of *Leishmania* from semen samples was shown to be possible ([35]; author’s personal communication), no study, to our knowledge, has assessed the persistence of viable *Leishmania* in semen after cryopreservation or the infectiousness of insemination. Therefore, as a safety precaution, canine semen can no longer be imported to New Caledonia, unless healthy dog donors have been tested seronegative for *Leishmania* by IFAT or Elisa or western blot at least 15 days after semen collection.

However, only 40 % of infected dogs show progression of the infection, in a typical sequence starting with the detection of PCR positivity in tissue aspirates followed by positive culture and then elevation of serological titre [36]. Consequently, the detection of infected but asymptomatic dogs before introduction into a leishmaniasis-free country remains a difficult challenge. When possible, as a serological test, western blot should be preferred because of its better sensitivity and specificity, and should be associated with kDNA real-time PCR on buffy-coat and lymph node and/or bone marrow aspirate, both tests repeated after 30 days to increase the likelihood of detection of subclinical dogs before importation.

Owners travelling with their animals from a Leishmaniasis-free country to any country where it is endemic should avoid mating their dogs (except with Leishmania-free partners) and protect them against sandfly bites with deltamethrin-impregnated collars or topical insecticides. The suitability of these collars and topics as a cost-effective and widely applicable method of dog protection has been well proven [37–39]. Although more data are still needed to confirm the efficiency of canine vaccines against *Leishmania infantum* infection by means of extensive field studies [36], vaccination might also be proposed for Caledonian autochthonous dogs prior to travel to endemic regions, on condition that vaccinated animals must be serologically and easily distinguishable from infected dogs.

## Conclusion

The first autochthonous canine leishmaniasis report in New Caledonia Island, known as a *Leishmania*-free territory, corroborates the existence of non-vectorial routes of leishmaniasis transmission. It supports the relevance of reinforced quarantine measures and the importance of careful vigilance among veterinary and medical clinicians even in *Leishmania*-free regions: the absence of travel history to endemic areas, or of known vectors in the residence country, should never be sufficient to exclude CanL as a possible diagnosis, because of the possibilities of transplacental, venereal, and direct dog-to-dog transmission.

Also, the occurrence of CanL cases within New Caledonia raises the debate of test requirements and diagnostic sensitivity prior to importation. Dogs imported from CanL- endemic regions should be tested twice with the most sensitive diagnostic screening techniques such as kqPCR coupled with western blot, since the risk of CanL endemicisation in New Caledonia cannot be ruled out, especially when a potential vector may be present.

Moreover, to reduce the risk of leishmaniasis infection of dogs travelling from New Caledonia to any country where it is endemic, we suggest owners should avoid mating their dogs and avoid direct dog-to-dog close prolonged contact. We recommend insecticide-impregnated collars or topical insecticides and vaccination as preventative control tools against the usual vector-borne form of leishmaniasis.

Finally, what we learned from the occurrence of this first autochthonous case in New Caledonia and the measures taken could be transposed to other leishmaniasis-free territories.

## Abbreviations

CanL: canine leishmaniasis
CIE: counter immunoelectrophoresis
CVBDs: canine vector-borne diseases
DASS-NC: Direction des Affaires Sanitaires et Sociales de Nouvelle Calédonie
ELISA: enzyme-linked Immunosorbent assay
IFAT: immunofluorescence antibody test
IU/ml: international unit per milliliter
kDNA: minicircle kinetoplast DNA
PCR: polymerase chain reaction
qPCR: quantitative real-time PCR
RPMI: Roswell Park Memorial Institute
RT-PCR: real-time PCR
WB: western blot
